# Entanglement Entropy of the Spin-1 Condensates at Zero Temperature

**DOI:** 10.3390/e20010080

**Published:** 2018-01-22

**Authors:** Zhibing Li, Yimin Liu, Wei Zheng, Chengguang Bao

**Affiliations:** 1 School of Physics, Sun Yat-Sen University, Guangzhou 510275, China; 2 Department of Physics, Shaoguan University, Shaoguan 510205, China; 3 Theory of Condensed Matter (T.C.M.) Group, Cavendish Laboratory, J.J.Thomson Avenue, Cambridge CB3 0HE, UK

**Keywords:** entanglement entropy, temperature zero, spin-1 Bose-Einstein condensates, 03.75.Kk, 03.65.Fd

## Abstract

For spin-1 condensates, the spatial degrees of freedom can be considered as being frozen at temperature zero, while the spin-degrees of freedom remain free. Under this condition, the entanglement entropy has been derived exactly with an analytical form. The entanglement entropy is found to decrease monotonically with the increase of the magnetic polarization as expected. However, for the ground state in polar phase, an extremely steep fall of the entropy is found when the polarization emerges from zero. Then the fall becomes a gentle descent after the polarization exceeds a turning point.

## 1. Introduction

Entanglement is a curiosity in quantum world. This topic is not only interesting in the academic aspect, but also possesses profound potential in application (e.g., in quantum communication) [1,2]. On the other hand, what happens at temperature (T) zero is one of the essential focuses in physical research. Although the T = 0 limit cannot be exactly realized, the related study pointing at this limit and its neighborhood is important (i.e., the superconductivity). There are already many findings related to this limit (like the famous Third Law of Thermodynamics). Accompanying the progress in technology, lower and lower temperature (i.e., <$10^{-10}$ K) can be realized. This progress definitely will attract more and more theoretical studies aiming at the neighborhood of T = 0. Furthermore, the technology for trapping the cold atoms is one of the most important progress in physics in recent years. This technology leads to the realization of man-made systems, namely, various Bose-Einstein condensates (BEC), and opens a very broad field of research promising in application [3,4,5,6,7,8]. Since these man-made systems are controllable, they can be used as analogs of other many-body systems to reveal the underlying physics. When T →0, the BEC will tend to its ground state (g.s.). With these in mind, this paper is devoted to studying the Von Neumann entanglement entropy (EE) of the g.s. of BEC.

Note that, in the g.s. of spin-0 BEC, the particles are not correlated, thus the entanglement is zero. However, for the g.s. of the spin-1 BEC, due to the spin-spin correlation, the entanglement is in general not zero. Previously, we have developed a tool, namely, the fractional percentage coefficients (FPC), for dealing with the complicated spin-states of the many-body systems with spin-1 particles [9,10]. With the FPC, one can extract the wave function of one (or more than one) particle(s) from the total wave function. Thus, the FPC works as an appropriate and powerful tool for studying the entanglement between a particle (or a few particles) and the others. Therefore, the g.s. of the spin-1 BEC are chosen to be studied in the follows. Due to the use of the FPC, the study is in an analytical way. The analytical formalism facilitates greatly related analysis.

## 2. Entanglement among a Particle and the Others

For the spin-1 BEC, the interaction can be written as $V=\Sigma_{i<j}\delta \left(r_{i}-r_{j}\right)\left(c_{0}+c_{2}F_{i}.F_{j}\right)$, where $F_{i}$ is the spin operator of the i-th particle. This interaction conserves the total spin *S* and its *Z*-component *M*. Therefore, a more precise theory for the g.s. should keep both *S* and *M* to be conserved. With this consideration the lowest state with the good quantum numbers *S* and *M* can be assumed as

(1) $$\Psi_{SM}=\Pi_{i=1}^{N}\varphi^{a}\left(r_{i}\right)\vartheta_{S,M}^{N}$$

where $\vartheta_{S,M}^{N}$ is the normalized all-symmetric total spin state with the given *S* and *M*, and *N* is the particle number, *S* is ranged from 0 to *N*. According to the theory given in [11], N−S must be even and the multiplicity is one (i.e., $\vartheta_{S,M}^{N}$ is unique for *S* and *M*). $\varphi^{a}\left(r_{i}\right)$ is the spatial wave function of the *i*-th particle. All the particles are condensed into this state which is most favorable for binding.

When *N* is large the general expression of $\vartheta_{S,M}^{N}$ is very complicated. For two particular cases S=0 and S=M=N, $\vartheta_{S,M}^{N}$ is given as an expansion in terms of the Fock-states [5]. Nonetheless, due to the introduction of the FPC, the explicit form of $\vartheta_{S,M}^{N}$ is irrelevant. One can extract a spin from $\vartheta_{S,M}^{N}$ as [9,10]

(2) $$\begin{array}{ccc} \vartheta_{SM}^{N} & = & \sum_{\mu }\chi_{\mu }\left(i\right)[a_{M\mu }^{NS}\vartheta_{S+1,M-\mu }^{N-1}\\ & & +b_{M\mu }^{NS}\vartheta_{S-1,M-\mu }^{N-1}] \end{array}$$

where $\chi_{\mu }\left(i\right)$ is the spin-state of the particle *i* in μ component, μ=0,±1. All the other particles are contained in $\vartheta_{S\pm 1,M-\mu }^{N-1}$, $a_{M\mu }^{NS}$ and $b_{M\mu }^{NS}$ are the FPC deived in [9,10], they appear as

(3) $$\begin{array}{ccc} a_{M\mu }^{NS} & = & \sqrt{\frac{[1+{\left(-1\right)}^{N-S}]\left(N-S\right)\left(S+1\right)}{2N(2S+1)}}C_{S+1,M-\mu ;1,\mu }^{SM}\\ b_{M\mu }^{NS} & = & \sqrt{\frac{[1+{\left(-1\right)}^{N-S}]S\left(N+S+1\right)}{2N(2S+1)}}C_{S-1,M-\mu ;1,\mu }^{SM} \end{array}$$

where $C_{S\pm 1,M-\mu ;1,\mu }^{SM}$ are the Clebsch-Gordan coefficients for spin-coupling.

We consider the *i*-th particle as the A-system, while the other particles as the B-system. Recall that the A-system contains three base functions $\chi_{\mu }\left(i\right)$, where μ=±1,0, and the B-system contains six base functions $\vartheta_{S\pm 1,M-\mu }^{N-1}$. Starting from Equation (2), it is straight forward to obtain the reduced density matrix

(4) $$\rho_{A}=\Sigma_{\mu }Z_{\mu }\left|\chi_{\mu }\left(i\right)><\chi_{\mu }\left(i\right)\right|$$

where

(5) $$Z_{\mu }={\left(a_{M\mu }^{NS}\right)}^{2}+{\left(b_{M\mu }^{NS}\right)}^{2}$$

From $\rho_{A}$, the EE is

(6) $$S_{ee}^{\left(1\right)}=-\Sigma_{\mu }Z_{\mu }\left(\ln Z_{\mu }\right)$$

## 3. Entanglement among a Pair of Particles and the Others

Using the FPC once again to extract one more spin from $\vartheta_{S\pm 1,M-\mu }^{N-1}$, Equation (2) can be rewritten as

(7) $$\vartheta_{SM}^{N}=\sum_{sS^{\prime }}h_{sS^{\prime }S}^{N}{\left[{\left(\chi \left(i\right)\chi \left(j\right)\right)}_{s}\vartheta_{S^{\prime }}^{N-2}\right]}_{S}$$

where the *i*-th and *j*-th spins in $\vartheta_{SM}^{N}$ have been extracted. The FPC are

(8) $$\begin{array}{ccc} h_{0,SS}^{N} & = & {\left[\left(N+S+1\right)\left(N-S\right)/\left(3N\left(N-1\right)\right)\right]}^{1/2}\\ h_{2,S+2,S}^{N} & = & {\left(\frac{\left(S+1)(S+2)(N-S)(N-S-2\right)}{\left(2S+1)(2S+3)N(N-1\right)}\right)}^{1/2}\\ h_{2,S,S}^{N} & = & {\left(\frac{S(2S+2)(N-S)(N+S+1)}{3(2S-1)(2S+3)N(N-1)}\right)}^{1/2}\\ h_{2,S-2,S}^{N} & = & {\left(\frac{S(S-1)(N+S+1)(N+S-1)}{\left(2S-1)(2S+1)N(N-1\right)}\right)}^{1/2} \end{array}$$

All the other $h_{sS^{\prime }S}^{N}$ are zero. Furthermore, when S=0, $h_{2,S,S}^{N}$ and $h_{2,S-2,S}^{N}$ should be zero.

We consider the *i*-th and *j*-th particles as the A-system, it contains six basis functions ${\left(\chi \left(i\right)\chi \left(j\right)\right)}_{s,m}$, where s=0 and 2, *m* is the Z-component of *s*. The others belong to the B-system, it contains fifteen basis functions $\vartheta_{S^{\prime },M-m}^{N-2}$, where $S^{\prime }=S\pm 2$ and *S*. The reduced density matrix is

(9) $$\begin{array}{ccc} \rho_{A} & = & \sum_{mS^{\prime }}|\sum_{s}X_{smS^{\prime }}{\left(\chi \left(i\right)\chi \left(j\right)\right)}_{s,m}>\\ & & <\sum_{s}X_{smS^{\prime }}{\left(\chi \left(i\right)\chi \left(j\right)\right)}_{s,m}| \end{array}$$

where $X_{smS^{\prime }}=h_{sS^{\prime }S}^{N}C_{sm;S^{\prime },M-m}^{SM}$. The matrix elements of $\rho_{A}$ with respect to the basis functions ${\left(\chi \left(i\right)\chi \left(j\right)\right)}_{s,m}$ is

(10) $${\left(\rho_{A}\right)}_{sm,s^{\prime }m^{\prime }}=\delta_{mm^{\prime }}\Sigma_{S^{\prime }}X_{smS^{\prime }}X_{s^{\prime }mS^{\prime }}$$

When m=±1 and ±2, both *s* and $s^{\prime }$ must equal to 2, thus only the diagonal matrix elements survive. However, when m=0, both *s* and $s^{\prime }$ can be 0 and 2. Accordingly, we have

(11) $$\begin{array}{ccc} {\left(\rho_{A}\right)}_{2,0;2,0} & = & {\left(X_{2,0,S+2}\right)}^{2}+{\left(X_{2,0,S}\right)}^{2}+{\left(X_{2,0,S-2}\right)}^{2}\equiv a\\ {\left(\rho_{A}\right)}_{2,0;0,0} & = & {\left(\rho_{A}\right)}_{0,0;2,0}=X_{2,0,S}X_{0,0,S}\equiv b\\ {\left(\rho_{A}\right)}_{0,0;0,0} & = & {\left(X_{0,0,S}\right)}^{2}\equiv c \end{array}$$

After a diagonalization, we obtain the two diagonal matrix elements as

(12) $$\lambda_{\pm }=\frac{1}{2}\left(a+c\pm \sqrt{{\left(a-c\right)}^{2}+4b^{2}}\right)$$

From the diagonal elements, the EE is

(13) $$S_{ee}^{\left(2\right)}=-\Sigma_{m}^{\prime }\left[{\left(\rho_{A}\right)}_{2m,2m}\ln{\left(\rho_{A}\right)}_{2m,2m}\right]-\lambda_{+}\ln\lambda_{+}-\lambda_{-}\ln\lambda_{-}$$

In the summation $\Sigma_{m}^{\prime }$, *m* runs over only ±1 and ±2.

## 4. Numerical Examples

**Figure 1:**
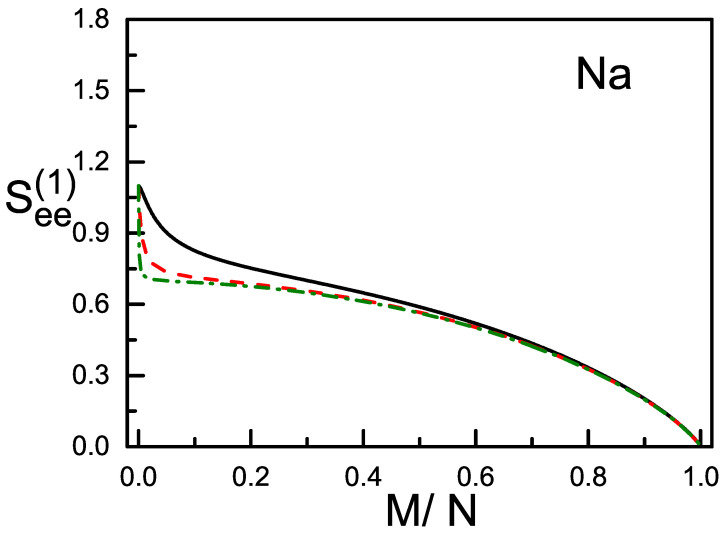
(color online) The entanglement entropy $S_{ee}^{\left(1\right)}$ of the g.s. in polar phase against M/N. The solid, dash, and dash-dot lines have *N* = 10^2^, 10^3^, and 10^4^, respectively.

**Figure 2:**
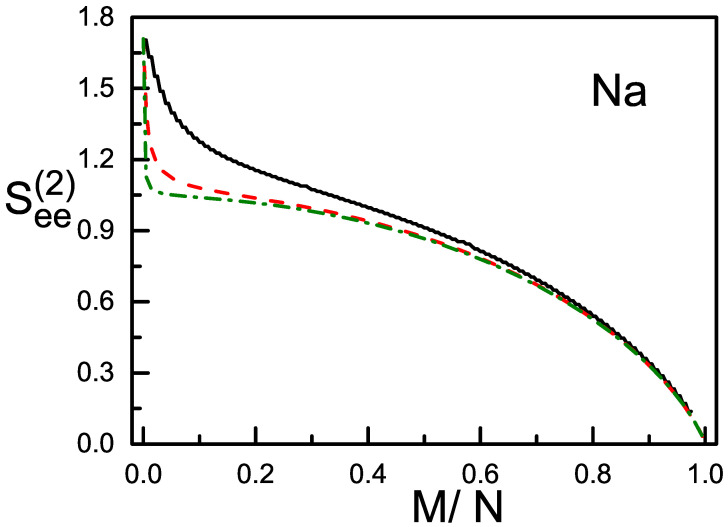
(color online) The entanglement entropy $S_{ee}^{\left(2\right)}$ of the g.s. in polar phase against M/N. The solid, dash, and dash-dot lines have *N* = 10^2^, 10^3^, and 10^4^, respectively.

**Figure 3:**
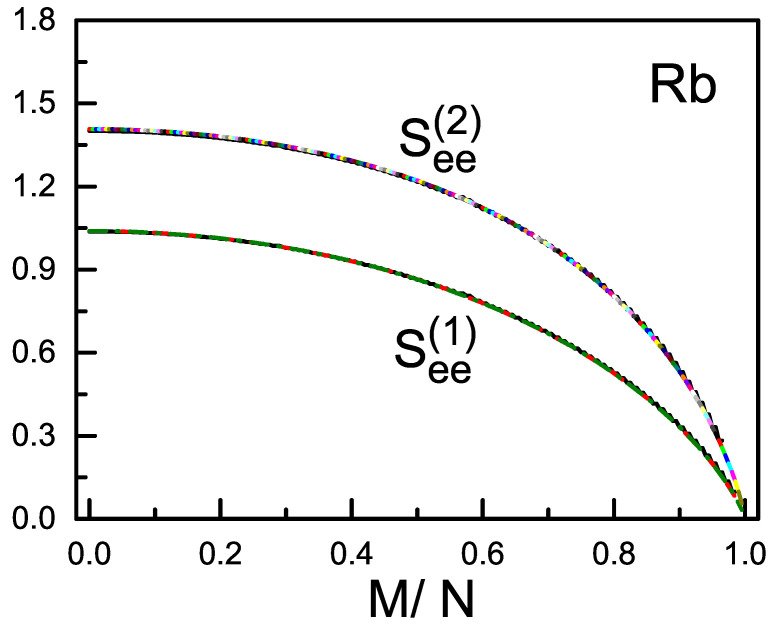
(color online) The entanglement entropy $S_{ee}^{\left(1\right)}$ and $S_{ee}^{\left(2\right)}$ of the g.s. in ferromagnetic phase against M/N. Curves for *N* = 10^2^, 10^3^, and 10^4^ are plotted. Dependence on *N* is not observable.

We consider the condensates with both the total spin *S* and its Z-component *M* being conserved. Thus the magnetic polarization M/N is conserved and depends on how the condensate is prepared. When $c_{2}>0$ (e.g., ^23^Na), a pair of atoms prefer to have their spins anti-parallel, and the total spin-states with a smaller *S* will have a lower energy. Accordingly, the g.s. has either S=0 (when the magnetization is zero, in this case the g.s. is purely composed of the s=0 pairs if *N* is even), or S=M (when the magnetization is non-zero). The g.s. $c_{2}>0$ is called to be in the polar phase. When *N* is given at 10^2^, 10^3^, and 10^4^, $S_{ee}^{\left(1\right)}$ and $S_{ee}^{\left(2\right)}$ of the g.s. in polar phase are plotted in Figure 1 and Figure 2, respectively, against M/N. On the other hand, when $c_{2}<0$ (e.g., ^87^Rb), a pair of atoms prefer to have their spins parallel, and the total spin-states with a larger *S* will have a lower energy. Accordingly, the g.s. has always S=N disregarding how the magnetization is. In this case the g.s. is in ferromagnetic phase. The $S_{ee}^{\left(1\right)}$ and $S_{ee}^{\left(2\right)}$ of the g.s. in ferromagnetic phase are plotted in Figure 3.

We found the following features (under the condition that the spatial degrees of freedom are frozen):

(i) In general $S_{ee}^{\left(2\right)}>S_{ee}^{\left(1\right)}$. In particular, when M/N=0 (zero polarization), for the g.s. in polar phase, $S_{ee}^{\left(1\right)}=\ln\left(3\right)=1.0986$; $S_{ee}^{\left(2\right)}\to -\left(\frac{2}{3}\ln\left(\frac{2}{15}\right)-\frac{1}{3}\ln\left(3\right)\right)=1.7095$ when N→∞. For the g.s. in ferromagnetic phase and when N→∞, $S_{ee}^{\left(1\right)}\to \frac{1}{2}\ln\left(8\right)=1.0397$ and $S_{ee}^{\left(2\right)}\to \frac{1}{8}\left[\ln\left(16\right)+4\ln\left(4\right)+3\ln\left(8\right)-3\ln\left(3\right)\right]=1.4075$. It is expected that, when more particles are included in the system-A (but ≤N/2), the EE would be larger. This is a point to be confirmed.

(ii) Both $S_{ee}^{\left(1\right)}$ and $S_{ee}^{\left(2\right)}$ decrease with the increase of polarization and they tend to zero when M/N→1 (fully polarized). When the condensate is fully polarized, all the spins are not free. Thus the vanish of the entanglement when M/N=1 is obvious.

(iii) $S_{ee}^{\left(1\right)}$ and $S_{ee}^{\left(2\right)}$ of the g.s. do not depend on the strengths of the interactions but only on the sign of the spin-dependent interaction (i.e., either attractive or repulsive. The former leads to the ferromagnetic phase while the latter leads to the polar phase). They also do not depend on the trap.

(iv) $S_{ee}^{\left(1\right)}$ and $S_{ee}^{\left(2\right)}$ depend in general weakly on *N*. This is particularly true for the ferromagnetic g.s. as shown in Figure 3, where the three curves for different *N* overlap nearly. However, for the g.s. in polar phase and when M/N increases from zero, a sudden fall of $S_{ee}^{\left(1\right)}$ and $S_{ee}^{\left(2\right)}$ is found. The fall is extremely steep when *N* is very large (e.g., $N\geq 10^{4}$) as shown in Figure 1 and Figure 2. It will become steeper when *N* becomes larger (compare the left parts of the dash and dash-dot curves in Figure 1 and Figure 2). There is a turning point, when M/N exceeds this point, the steep fall will be replaced by a gentle descent and tends to zero as mentioned. Usually, the condensates have a large *N*. Thus, for the polar g.s., there is a narrow and highly sensitive region when the polarization increases from zero. In this region the structure of the g.s. (measured by the ratios of the numbers of *μ*-atoms $N_{\mu }$) is highly sensitive to the polarization (refer to Figure 3 of the ref. [12]). In fact, when S=M and by using Equation (2), it is straight forward to obtain that all three $\frac{d}{dS}\left(N_{\mu }\right)\propto 1/{\left(2S+3\right)}^{2}$. Recall that, when S=M and *N* is large, a small increase in the polarization will cause a large increase in *S*. Thus the factor $1/{\left(2S+3\right)}^{2}$ leads to the appearance of the turning point.

## 5. Discussions and Final Remarks

General expressions for the bi-partite entanglement entropies of one atom and the others, as well as a pair of atoms and the others, in the ground state of spin-1 BEC are derived. Numerical examples for the condensates in polar phase and ferromagnetic phase are given. It is shown that, when the condensate has a large *N*, the two entanglement entropies of the g.s. in polar phase drops sharply when M/N increases from zero. On the other hand, the two entanglement entropies of the g.s. in ferromagnetic phase are almost independent of the atom number and decreases smoothly with M/N. Note that, when S=0, the total spin-state is purely composed of the s=0 pairs (if *N* is even, otherwise, one particle is not in a pair). While when *S* increases, more and more s=2 pairs will mix in. For the polar g.s. with a large *N*, a small increase in M/N will lead to a big increase in *S* (because S=M) and therefore cause a great change in spin-structure (refer to the $1/{\left(2S+3\right)}^{2}$ law mentioned above). This responds to the big fall appearing in Figure 1 and Figure 2 when $N=10^{4}$. Whereas for the ferromagnetic g.s., the increase in M/N will not change *S* (because *S* remains =N). Thus the structure of the total spin-state remains unchanged but only its geometry. This responds to the smooth and monotonic variation as shown in Figure 3.

Note that, when only spin-degrees of freedom are concerned, both Equations (6) and (13) are exact analytical expressions for the EE of the systems. For spin-1 atoms, the spin-independent interaction is nearly two order stronger than the spin-dependent interaction. Therefore the spatial excitation energy would be in general much higher than the spin-excitation energy. Accordingly, when the temperature is sufficiently low, the spatial excitation would not be involved and the spatial degrees of freedom could be considered as being frozen [13]. In this case, the EE are exactly given by the above two formulae.

The entanglement of many-body systems is tightly connected to the characteristics of phase diagram and other thermodynamic quantities [14,15]. The present paper has concentrated on the entanglement of internal degrees of freedom of the system in the zero temperature limit. Exploring the effect of spin-orbital coupling, including spatial excitations, would be interesting future work.
